# Micrometeorological monitoring reveals that canopy temperature is a reliable trait for the screening of heat tolerance in rice

**DOI:** 10.3389/fpls.2024.1326606

**Published:** 2024-02-16

**Authors:** Wentao Tian, Qilin Mu, Yuan Gao, Yunbo Zhang, Yi Wang, Shuangcheng Ding, Kelvin Dodzi Aloryi, Nnaemeka Emmanuel Okpala, Xiaohai Tian

**Affiliations:** ^1^ Hubei Collaborative Innovation Center for Grain Industry, Yangtze University Jingzhou, Hubei, China; ^2^ Engineering Research Center of Ecology and Agricultural Use of Wetland, Ministry of Education, Hubei, China

**Keywords:** climate change, rice, heat stress, weather scenario, spikelet fertility, canopy temperature, stomatal conductance

## Abstract

Micrometeorological monitoring is not just an effective method of determining the impact of heat stress on rice, but also a reliable way of understanding how to screen for heat tolerance in rice. The aim of this study was to use micrometeorological monitoring to determine varietal differences in rice plants grown under two weather scenarios−Long-term Heat Scenario (LHS) and Normal Weather Scenario (NWS)− so as to establish reliable methods for heat tolerance screening. Experiments were conducted with two heat susceptible varieties−Mianhui 101 and IR64−and two heat tolerant varieties, Quanliangyou 681 and SDWG005. We used staggered sowing method to ensure that all varieties flower at the same time. Our results showed that heat tolerant varieties maintained lower canopy temperature compared to heat susceptible varieties, not just during the crucial flowering period of 10 am to 12 pm, but throughout the entire day and night. The higher stomatal conductance rate observed in heat tolerant varieties possibly decreased their canopy temperatures through the process of evaporative cooling during transpiration. Conversely, we found that panicle temperature cannot be used to screen for heat tolerance at night, as we observed no significant difference in the panicle temperature of heat tolerant and heat susceptible varieties at night. However, we also reported that higher panicle temperature in heat susceptible varieties decreased spikelet fertility rate, while low panicle temperature in heat tolerant varieties increased spikelet fertility rate. In conclusion, the results of this study showed that canopy temperature is probably the most reliable trait to screen for heat tolerance in rice.

## 1 Introduction

One of the greatest threats facing rice breeders and producers is the increase in temperature, which could be attributed to climate change (Okpala et al., 2021). According to Lu et al. (2014) temperature affects rice germination, growth, tiller number, heading time and yield. Shi et al. (2017) reported that heat stress as a result of high day and night temperature led to decrease in rice yield. Studies conducted in the past six decades by Satake and Yoshida (1978); Matsui et al. (1997); Matsui and Omasa (2002); Prasad et al. (2006), have shown that rice is very susceptible to heat during heading and flowering. Tian et al. (2009) demonstrated that three consecutive days of heat waves led to visible heat damage in rice. Okpala et al. (2020) and Okpala et al. (2021) have shown that high temperature at the onset of heading through maturity had negative impact on rice grain quality. Perdomo et al. (2015) and Yamori et al. (2011) showed that temperature had large effects on the photosynthetic components of rice and total plant biomass. Dillahunty et al. (2001) stated that exposure to high temperature above 50°C for over 12 h has been shown to cause colour degradation in freshly harvested rice grains. Li et al. (2020) and Zhang et al. (2018), reported that heat stress caused more damage in heat susceptible rice varieties, compared to heat tolerant rice varieties.

Sun et al. (2022) modeled rice growth under heat stress at the flowering and filling stages with two heat stress models into the CERES-Rice model and found that in the coming years, heat stress will decrease rice yield in China. Other studies have shown that rice production in the middle and lower reaches of the Yangtze River−which accounts for about two thirds of the country’s rice production area−is constantly and seriously affected by heat (Wassman et al., 2009; Tian et al., 2010; Teixeira et al., 2013; He et al., 2018; Dou et al., 2019). It has been estimated by MODIS and other models that 5.7×10^6^ ha or 35.9% of the total rice area in Yangtze River basin was damaged as a result of heat (Dou et al., 2019). The effect of summer temperatures on rice growth and development ranges from moderate (T_mean_≥30°C for more than three consecutive days) to severe (T_mean_≥30°C for more than five consecutive days) heat stress (Tian et al., 2009).

However, the occurrence of heat damage is not solely due to temperature, but also due to complex micrometeorological factors in the field−such as, humidity, wind speed, and sunlight intensity−which can affect the temperature of plant tissues and their surroundings (Tian et al., 2010; Siebert et al., 2014; Sathishraj et al., 2016). According to the findings of Matsui et al. (2007) rice that headed and flowered in temperatures above 40°C in New South Wales, Australia, were rarely affected by the heat due to the lower relative humidity and higher wind speed in the area. These two factors probably led to strong transpiration cooling, which might reduce the rice plant body and ambient temperature by 7°C, compared to the air temperature, thus preventing damage. Tian et al. (2010) reported that high temperatures during rice growth at the Yangtze River basin were accompanied by high humidity and low wind speeds. Therefore, rice was prone to damage when daily average temperatures are ≥30°C or daily maximum temperatures are ≥35°C. Tian et al. (2010) also reported that heat stress increased panicle temperature in heat susceptible rice varieties by 1–3°C, compared to the air temperature at the onset flowering. Other studies have shown that heat stress also occurs in the tropics, with similar environmental conditions to the Yangtze River basin, but with lower relative humidity (Ishimaru et al., 2015; Sathishraj et al., 2016).

The impact of high temperature on rice varies from variety to variety (Okpala et al., 2021). The pioneering work of Satake and Yoshida (1978) revealed that rice varieties differed in their response to heat stress by 4–5°C. Subsequent studies have also shown varietal differences in response to heat growth conditions (Prasad et al., 2006; Maruyama et al., 2013; Ishimaru et al., 2015; Shi et al., 2015; Okpala et al., 2020). The reason for these varietal differences as a result of heat stress in rice has not been fully established. However, report by Gong et al. (2023) showed that varietal differences in response to heat stress in rice were associated with stomatal conductance and canopy temperature. Previous studies conducted with wheat and cotton, also showed that varietal differences in response to heat stress were associated to canopy temperature and stomatal conductance (Lu et al., 1994; Lu et al., 1998; Ayeneh et al., 2002; Lopes and Reynolds, 2010; Conaty et al., 2015).

Heat damage has been observed in rice when maximum daily temperatures reached 33–35°C (Satake and Yoshida, 1978; Matsui et al., 1997; Jagadish et al., 2010), with the degree of damage closely related to the duration of exposure prior to and during heading (Tian et al., 2009; Sathishraj et al., 2016). Since the actual temperature of the flower organs more accurately reflect the impact of heat stress, many studies have focused on panicle and canopy temperatures to characterise rice heat damage in the field (Takai et al., 2010; Tian et al., 2010; Siebert et al., 2014; Zhang et al., 2016; Sathishraj et al., 2016). However, most of the studies focused on varietal difference during the crucial flowering period of 10 am to 12 pm in rice. Therefore, there is need to conduct extensive research that would monitor the micrometeorology of rice, not just during the crucial flowering hours of 10 am to 12 pm, but throughout the entire day and night. Therefore, the aim of this study was use to micrometeorological monitoring to determine varietal differences in rice grown under two weather scenarios−normal weather scenario (NWS) and long-term heat scenario (LHS)−in the middle reaches of the Yangtze River in China and to establish key micrometeorological variables for screening of heat tolerance in rice.

## 2 Materials and methods

### 2.1 Experimental site, plant materials and management

This study was conducted at the experimental farm of Yangtze University, located in the middle reaches of the Yangtze River basin. We used four *indica* rice varieties− Mianhui101 (MH101), IR64, Quanliangyou 681 (QLY681) and SDWG005−with contrasting heat tolerance levels for the investigations (**Table 1**). MH101 and IR64 are heat susceptible varieties, while QLY681 and SDWG005 are heat tolerant varieties.

**Table 1:** Varietal information and adjusted sowing dates under the staggered planting system. ‡, and † represent the heat tolerant and heat-susceptible varieties, respectively.

Variety	Type	Batch 1	Batch 2	Batch 3	Batch 4
Mianhui101^†^	*Indica*/inbred	28-Apr	8-May	18-May	28-May
IR64^†^	*Indica*/inbred	21-Apr	1-May	11-May	21-May
Quanliangyou681^‡^	*Indica*/hybrid	25-Apr	5-May	15-May	25-May
SDWG005^‡^	*Indica*/inbred	26-Apr	6-May	15-May	26-May

The soil texture at the experimental site was clay loam, with 18.3g/kg organic matter, 1.14g/kg hydrolysable N, 34.25 mg/kg available P, and 105.13 mg/kg available K.

Staggered sowing method was used for the experiment and it was done in four different batches: Batch 1, Batch 2, Batch 3 and Batch 4, as shown in **Table 1**. Staggered sowing method was used so as to have all four rice varieties flower at the same time. Based on the available phenological data, we determined the sowing dates of each of the four varieties.

The experiment was conducted in randomised blocks and the plot area was 2×3 m^2^. Pre-germinated rice seeds were sown in seedbeds. After 23 days, rice seedlings were transplanted to the farming field. Each hill has 3 to 4 seedlings, with a hill spacing of 0.20 by 0.30 m. A day before transplanting, compound fertiliser (N:P:K=22:8:12, 150kg/1000m^2^) was applied in the entire planting field.

### 2.2 Crop ambient temperature and humidity

The meteorological data used in the study were obtained from two weather stations, as the ambient meteorological variables of the rice plants were used as reference to the data we recorded in the canopy and plant surfaces. The first station (HOBO-U30, ONSET) was set up at the edge of the experimental farm, with sensors set 2 m above the ground and hereafter referred to as the “above the canopy”. The second one was from a nearby (500 m) weather station, Jingzhou Agricultural Meteorological Station, which is hereafter referred to as the “open space”. Open space temperature and ambient temperature are used interchangeably in this article.

### 2.3 Heat stress based on two weather scenarios

Heat stress was based on two weather scenarios−Normal Weather Scenario (NWS) and Long-term Heat Scenario (LHS)−at the experimental farm of Yangtze University (**Table 2**).

**Table 2:** Classification of the weather scenarios used in the experiment.

Weather scenario	Criteria
Normal Weather Scenario (NWS)	Sustained ≥3 days, with daily mean temperature <30°C or daily maximum temperature <35°C
Long-term Heat scenario (LHS)	Sustained ≥3 days, with daily mean temperature ≥30°C or daily maximum temperature ≥35°C

### 2.4 Rice heading date

Heading date was based on when 10 mm of the panicle tip had already emerged from the flag leaf sheath of the rice plant. At the onset of heading, the percentage of rice panicles that emerged from the rice sheath was recorded daily at 5 pm. In other to determine the percentage of panicle emergence in each plot, we randomly selected twenty rice plant hills.

### 2.5 Leaf and panicle surface temperature

Leaf and panicle temperature were measured in rice plants that had almost the same growth stage. We identified rice plants with almost the same growth stage by marking their fluorescence at the onset of heading. Afterwards, we randomly selected five marked rice plants in each plot for leaf and panicle surface temperature measurements using an infrared thermometer (MI-230, Apogee Instruments). The leaves and panicles of the rice plants were measured each day at 10:00, 14:00 and 20:00 hours, which coincided with flowering, at the highest temperature of the day, and in the evening, respectively. In other to determine the differences under various regimes, we identified 9^th^ August to be the last flowering day for the LHS and 26^th^ August as the last flowering day for the NWS.

### 2.6 Canopy temperature and humidity

**Figure 1:**
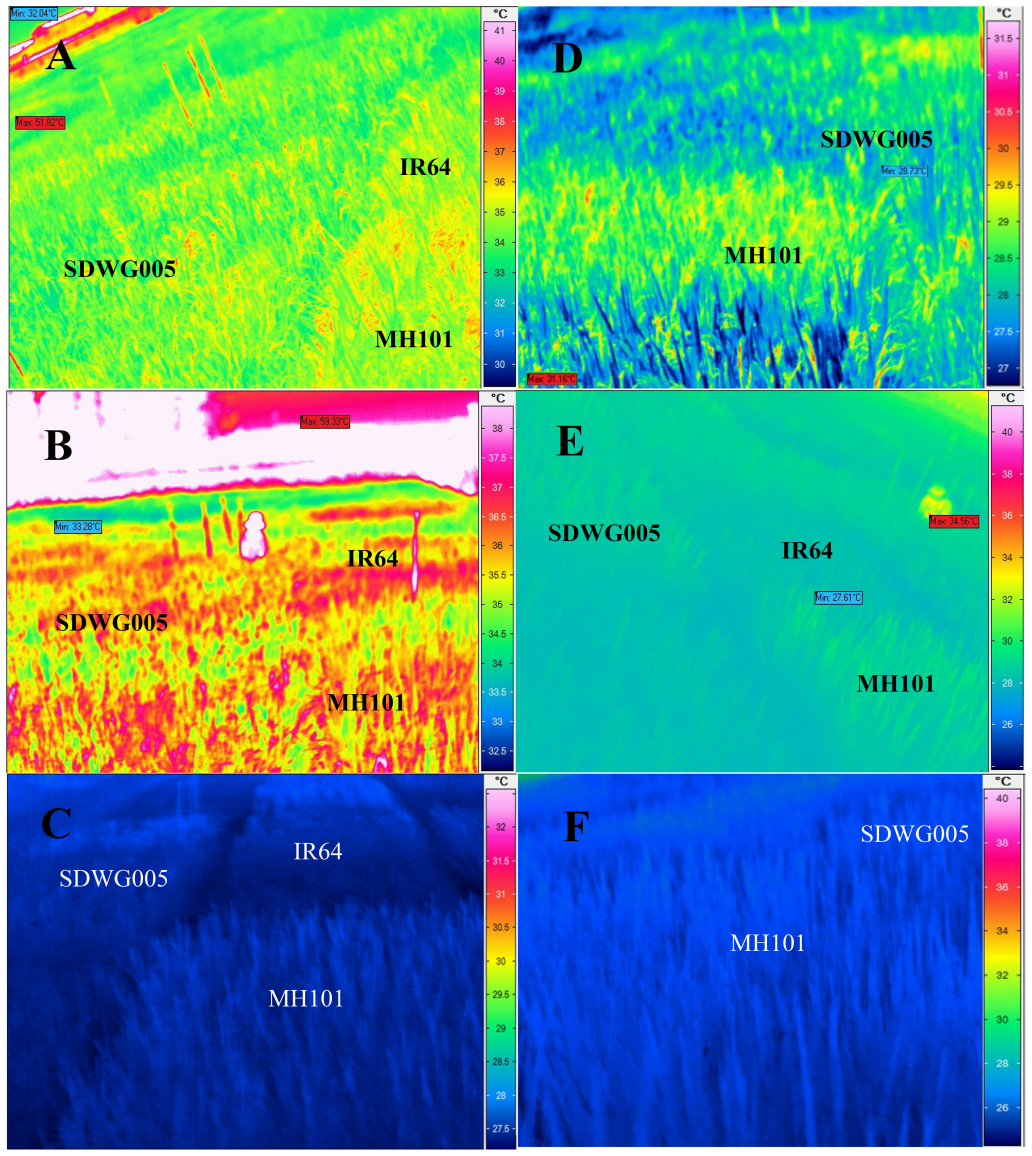
Infrared thermal photos of the canopy surface taken at critical time of the day (10 am, 2 pm and 8 pm respectively) during the flowering stage in field-grown rice, showing differences in canopy surface temperature between the typical varieties under **(A–C)** the long-term heat scenario (LHS) and **(D–F)** the normal weather scenario (NWS) at **(A, D)** 10:00, **(B, E)** 14:00, and **(C, F)** 20:00.

Canopy temperature and humidity were measured by placing Micrometeorological Instrument for the Near-Canopy Environment of Rice (MINCER)—invented by the Institute of Agricultural Environmental Science, Japan (Yoshimoto et al., 2011)—in the middle of each plot, with the sensor targeting the middle position of the panicle (**Figure 1**). Canopy temperature and humidity were recorded every 3 minutes.

### 2.7 Stomatal conductance

Stomatal conductance were measured daily with a steady-state porometer (SC-1, Decagon) at 10:00 and 16:00 hours. We measured stomatal conductance by randomly selecting five flag leaves that emerged almost at the same time (determined by the fluorescence marks).

### 2.8 Spikelet fertility

Spikelet fertility was estimated using the procedures of Prasad et al. (2006). The ratio of filled grains to the total number of reproductive sites (florets) was estimated based on stipulations of Yan et al. (2017) and are expressed in percentage.

### 2.9 Germinated pollens shed on the stigma

Germinated pollen shed on the stigma was determined using the procedures of Matsui and Kagata (2003). Twenty spikelets were randomly sampled in each plot at 13:00 on the first, third, and fourth days after flowering under NWS and LHS.

### 2.10 Thermal imaging of the canopy surface

Thermal imaging of the canopy surface was carried out at the same time that panicle and leaf temperature was determined. Thermal imaging was determined using A FLIR ThermaCAMTM S65 system (FLIRSystems Inc., Portland, OR., USA), with a wide-angle camera lens (18 mm IR-LENS). The camera was set up 0.5 m away from the rice plants. The data were analysed with ThermaCAM Researcher Pro 2.7 software (FLIR Systems Inc.).

### 2.11 Statistical analysis

Data were analysed with DPS 7.5. Tukey’s Least significant difference (LSD) were determined at the probability levels of 5% and 1% between treatments.

## 3 Results

### 3.1 Flowering and weather scenario coincidence

**Figure 2:**
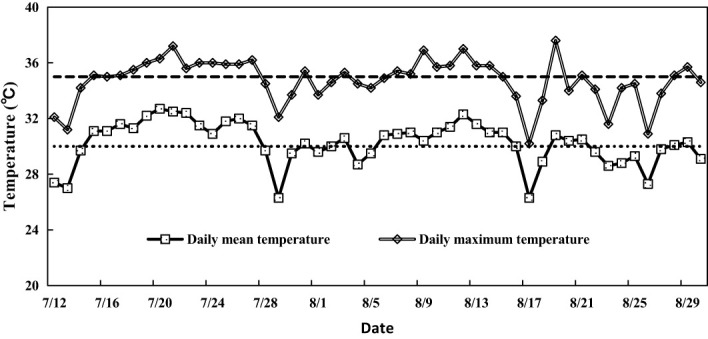
Daily course of temperature during reproductive growth in rice in 2018 at the experimental site. The dotted lines show the tentative critical temperatures—30°C for daily mean temperature and 35°C for daily maximum temperature. The X-axis is mm/dd.

**Table 3** shows the period when flowering in all four varieties coincided with the two weather scenarios used in this study. Flowering coincided with LHS from 6^th^ August to 9^th^ August, while it coincided with NWS from 24^th^ to 26^th^ August. **Table 2** shows the criteria used in determining LHS and NWS. **Figure 2** shows the daily main and daily maximum temperature during the reproductive phase of the rice varieties used in this study.

**Table 3:** Flowering periods of the four rice varieties under the staggered planting system that.

Variety	Batch 1	Batch 2	Batch 3	Batch 4
Mianhui101	28-July–1-Aug	6-Aug–9-Aug	15-Aug–16-Aug	24-Aug–26-Aug
IR64	6-Aug–9-Aug	15-Aug–16-Aug	24-Aug–26-Aug	29Aug–2-Sept
Quanliangyou681	25-July–29-July	6-Aug–9-Aug	15-Aug–16-Aug	24-Aug–26-Aug
SDWG005	27-July–31-July	6-Aug–9-Aug	15-Aug–16-Aug	24-Aug–26-Aug

### 3.2 Spikelet fertility and germinated pollen in the stigma under different weather scenarios

#### 3.2.1 Spikelet fertility

As shown in **Table 4**, we observed that increase in heat stress led to significant decrease in spikelet fertility. Generally in all varieties used in this study, our findings showed that spikelet fertility decreased the most in LHS, compared to NWS. Mianhui 101 had spikelet rate of 80.9 ± 10.4 and 65.4 ± 5.1 for NWS and LHS, respectively. IR64 had spikelet rate of 85.9 ± 3.7 and 66.3 ± 10.1 for NWS and LHS, respectively. Quanliangyou 681 had spikelet fertility of 88.8 ± 2.9 and 80.9 ± 7.4 for NWS and LHS, respectively. Finally, SDWG005 had spikelet fertility of 77.7 ± 8.9 and 74.3 ± 9.8, for NWS and LHS, respectively.

**Table 4:** Grain setting rate of the four rice varieties under the two weather scenarios.

Variety	Grain setting rate (%)	
	LHS	NWS
Mianhui101	65.4 ± 5.1c^**^	80.9 ± 10.4b
IR64	66.3 ± 10.1c^**^	85.9 ± 3.7a
Quanliangyou681	80.9 ± 7.4a^*^	88.8 ± 2.9a
SDWG005	74.3 ± 9.8b	77.7 ± 8.9c

** and * indicate significant differences at P<0.01 and P<0.05, respectively, between a variety in the long-term heat scenario (LHS) and the same variety under the normal weather scenario (NWS).

Different lowercase letters indicate significant differences between varieties under the same weather scenario at the 5% level using Tukey’s test (n=20).

We observed that in NWS, Quanliangyou 681 had the highest spikelet fertility of 88.8 ± 2.9%, followed by IR64 with spikelet fertility of 85.9 ± 3.7%. Conversely, SDWG005 had the lowest spikelet fertility of 77.7 ± 8.9% in NWS, followed by Mianhui 101 with spikelet fertility of 80.9 ± 10.4%.

Our findings showed that the impact of heat on spikelet fertility varied among the varieties. In heat tolerant variety, SDWG005, we observed that increase in temperature had no significant difference in the spikelet fertility between NWS and LHS. However, in the second heat tolerant variety, Quanliangyou 681, we observed significant difference−with Pvalue <0.05−in spikelet fertility between NWS and LHS. In heat susceptible varieties, Mianhui 101 and IR64, we found significant difference−with Pvalue <0.01−in the spikelet fertility of NWS compared to the spikelet fertility of LHS in each variety. Our results also showed that heat susceptible varieties, Mianhui 101 and IR64, had significantly lower spikelet fertility, compared to heat tolerant varieties, QLY681 and SDWG005, grown under LHS.

#### 3.2.2 Pollen germination rate

In **Table 5** we showed the pollen germination rate on the anther of the rice varieties used in the study. In Mianhui 101 we observed that the pollen germination rate on each anther were 42.1 ± 5.4% and 36.7 ± 5.0 for NWS and LHS, respectively. The pollen germination in IR64 were 50.0 ± 6.9 and 37.2 ± 5.0 for NWS and LHS, respectively. In Quanliangyou 681 we reported the pollen germination rate of 57.0 ± 4.1 and 51.4 ± 7.5 for NWS and LHS, respectively. Finally, in SDWG005 we reported the pollen germination rate of 41.8 ± 15.2 and 37.5 ± 4.9 for NWS and LHS, respectively.

**Table 5:** Pollen germination rate on each anther of the four rice varieties under the two weather scenarios.

Variety	Pollen germination rate (%)	
	LHS	NWS
Mianhui101	36.7 ± 5.0b^**^	42.1 ± 5.4c
IR64	37.2 ± 5.0b^**^	50.0 ± 6.9b
Quanliangyou681	51.4 ± 7.5a^*^	57.0 ± 4.1a
SDWG005	37.5 ± 4.9b	41.8 ± 15.2c

** and * indicate significant differences at P<0.01 and P<0.05, respectively, between a variety in the long-term heat scenario (LHS) and the same variety under the normal weather scenario (NWS).

Different lowercase letters indicate significant differences between varieties under the same weather scenario at the 5% level using Tukey’s test (n=20).

Our finding showed that in NWS, Quanliangyou 681 had the highest percentage of pollen germination (57.0 ± 4.1%), followed by IR64 with 50.0 ± 6.9%. SDWG005 had lowest percentage of pollen percentage (41.8 ± 15.2%), followed by Mianhui 101 with 42.1 ± 5.4%.

The impact of heat on pollen germination in the four varieties varied drastically. The results of our statistical analysis showed that in heat tolerant variety, SDWG005, heat stress had no significant impact in the pollen germination of rice grown in NWS compared to LHS. In the second heat tolerant variety, Quanliangyou 681, we observed significant difference−with Pvalue <0.05−in rice grown in NWS compared with LHS. However, in heat susceptible varieties, Mianhui 101 and IR64, we observed that heat had profound impact on the pollen germination rates. In Mianhui 101 we observed significant difference−with Pvalue <0.01−in the pollen germination rate of rice grown in NWS compared to LHS. In IR64 our findings showed significant difference−with Pvalue <0.01−in the pollen germination rate of rice grown in NWS compared to LHS.

### 3.3 Leaf and panicle temperature monitored during flowering under different weather scenarios

Our results as shown in **Table 6** reveal that at 10:00 and 14:00, SDWG005 and Quanliangyou681−heat-tolerant varieties grown under LHS−had significantly lower leaf and panicle temperatures compared to Mianhui 101 and IR64 which are heat susceptible varieties.

**Table 6:** Leaf and panicle temperatures at 10 am, 2 pm and 8 pm during flowering under LHS.

Time	Variety	Temperature (°C)		
		Leaf	Panicle	Leaf–panicle
6 August				
10 am	Mianhui101	31.5 ± 0.9a	32.8 ± 0.5a**	–1.3a
	IR64	31.4 ± 0.3a	32.5 ± 0.1a**	–1.1a
	Quanliangyou681	31.3 ± 0.4a	32.0 ± 0.4b**	–0.7b
	SDWG005	31.2 ± 0.9a	31.9 ± 0.8b**	–0.8b
2 pm	Mianhui101	31.8 ± 0.1a	33.2 ± 0.5a**	–1.6a
	IR64	31.7 ± 0.2a	32.8 ± 0.4a**	–1.1b
	Quanliangyou681	31.3 ± 0.7b	32.1 ± 0.5b**	–0.8c
	SDWG005	31.2 ± 0.1b	32.1 ± 0.7b**	–0.9c
8 pm	IR64	27.8 ± 0.1a	27.7 ± 0.3a	0.1b
	Mianhui101	28.2 ± 0.4a	27.9 ± 0.8a	0.3a
	Quanliangyou681	28.3 ± 0.4a	27.9 ± 0.4a	0.4a
	SDWG005	27.8 ± 0.7a	27.6 ± 0.9a	0.2a
8 August				
10 am	Mianhui101	31.5 ± 0.8a	32.4 ± 0.7a**	–0.8a
	IR64	31.3 ± 0.6a	32.2 ± 0.6a**	–0.7a
	Quanliangyou681	30.8 ± 0.9b	31.4 ± 0.6b**	–0.5b
	SDWG005	30.9 ± 0.4b	31.6 ± 0.6b**	–0.6b
2 pm	Mianhui101	33.5 ± 0.4a	33.7 ± 0.4a**	–0.2c
	IR64	32.6 ± 0.3b	33.1 ± 0.6b**	–0.5b
	Quanliangyou681	31.6 ± 0.4c	32.8 ± 0.6c**	–1.1a
	SDWG005	31.8 ± 0.8c	32.9 ± 0.5c**	–1.1a
8 pm	Mianhui101	25.7 ± 0.3a	25.6 ± 0.2a	0.1a
	IR64	25.5 ± 0.2a	25.4 ± 0.2a	0.1a
	Quanliangyou681	25.8 ± 0.1a	25.6 ± 0.2a	0.2a
	SDWG005	25.5 ± 0.3a	25.4 ± 0.3a	0.2a
9 August				
10 am	Mianhui101	31.5 ± 0.8a	32.4 ± 0.7a**	–0.8a
	IR64	31.3 ± 0.6a	32.2 ± 0.6a**	–0.7a
	Quanliangyou681	30.8 ± 0.9b	31.4 ± 0.6b**	–0.5b
	SDWG005	30.9 ± 0.4b	31.6 ± 0.6b**	–0.6b
2 pm	Mianhui101	33.5 ± 0.4a	33.7 ± 0.4a**	–0.2c
	IR64	32.6 ± 0.3b	33.1 ± 0.6b**	–0.5b
	Quanliangyou681	31.6 ± 0.4c	32.8 ± 0.6c**	–1.1a
	SDWG005	31.8 ± 0.8c	32.9 ± 0.5c**	–1.1a
8 pm	Mianhui101	25.7 ± 0.3a	25.6 ± 0.2a	0.1a
	IR64	25.5 ± 0.2a	25.4 ± 0.2a	0.1a
	Quanliangyou681	25.8 ± 0.1a	25.6 ± 0.2a	0.2a
	SDWG005	25.5 ± 0.3a	25.4 ± 0.3a	0.2a

** and * indicate significant differences at P<0.01 and P<0.05, respectively, between panicle and leaf temperatures within a variety.

Different lowercase letters indicate significant differences between varieties at the P<0.05 level using Tukey’s test (n=20).

During the period that LHS coincided with flowering; we observed higher panicle temperatures compared to leaf temperature in all four varieties at 10:00 and 14:00. However, at 20:00 we observed slightly higher leaf temperatures−that’s not statistically significant−compared to panicle temperatures in all four rice varieties as shown in **Table 6**. Our results showed that at 10:00 and 14:00, Mianhui 101 and IR64 which are heat susceptible varieties, had significantly higher leaf and panicles temperatures, compared to SDWG005 and Quanliangyou 681 which are heat-tolerant varieties. We observed that at 20:00 the leaf and panicles temperatures of both the heat-tolerant and heat susceptible varieties were not significantly different.

However, when NWS coincided with flowering as shown in **Table 7**; our findings showed mostly significantly higher panicle temperature compared to leaf temperature in all four varieties at 10:00 and significantly higher leaf temperatures compared to panicle temperature at 20:00. We observed that the difference between leaf and panicle temperature at 14:00 were mostly not significant. We equally observed that at 10:00, 14:00 and 20:00, leaf and panicle temperatures of all four varieties−both the heat-tolerant and heat intolerant varieties−were not significantly different.

**Table 7:** Leaf and panicle temperatures at 10 am, 2 pm and 8 pm during flowering under NWS.

Time	Variety	Temperature (°C)		
		Leaf	Panicle	Leaf–panicle
24 August				
10 am	Mianhui101	26.9 ± 0.7a	27.6 ± 0.7a**	–0.7b
	IR64	27.3 ± 0.7a	28.4 ± 0.1a**	–1.1a
	Quanliangyou681	27.2 ± 0.3a	27.9 ± 0.9a**	–0.7b
	SDWG005	27.5 ± 0.4a	28.1 ± 0.4a**	–0.6b
2 pm	Mianhui101	29.1 ± 0.6a	29.5 ± 0.8a**	–0.4a
	IR64	28.7 ± 0.6a	29.1 ± 0.2a**	–0.4a
	Quanliangyou681	28.8 ± 0.6a	29.1 ± 0.9a	–0.3a
	SDWG005	28.9 ± 0.6a	29.2 ± 0.7a	–0.3a
8 pm	Mianhui101	25.3 ± 0.6a	24.1 ± 0.1a**	1.2a
	IR64	25.3 ± 0.6a	24.2 ± 0.3a**	1.1a
	Quanliangyou681	25.3 ± 0.6a	24.3 ± 0.2a**	1.0a
	SDWG005	25.2 ± 0.6a	24.1 ± 0.2a**	1.1a
26 August				
10 am	Mianhui101	26.1 ± 0.6a	26.9 ± 0.7a**	–0.8a
	IR64	25.8 ± 0.5a	26.8 ± 0.3a**	–0.9a
	Quanliangyou681	25.7 ± 0.6a	26.5 ± 0.7a**	–0.8a
	SDWG005	26.1 ± 0.4a	26.8 ± 0.5a**	–0.7a
2 pm	Mianhui101	26.9 ± 0.8a	27.1 ± 0.3a	–0.2a
	IR64	26.2 ± 0.9a	27.1 ± 0.2a	–0.4a
	Quanliangyou681	26.7 ± 0.5a	26.9 ± 0.7a	–0.2a
	SDWG005	26.6 ± 0.3a	27.0 ± 0.5a	–0.4a
8 pm	Mianhui101	25.7 ± 0.8a	24.5 ± 0.7a**	1.2a
	IR64	25.4 ± 0.6a	24.6 ± 0.1a**	0.8b
	Quanliangyou681	25.7 ± 0.2a	24.6 ± 0.5a**	1.1a
	SDWG005	25.5 ± 0.5a	24.5 ± 0.7a**	1.0a

** and * indicate significant differences at P<0.01 and P<0.05, respectively, between panicle and leaf temperatures within a variety.

Different lowercase letters indicate significant differences between varieties at the P<0.05 level using Tukey’s test (n=20).

### 3.4 Canopy temperature and humidity monitored during flowering under different weather scenarios

**Figure 3:**
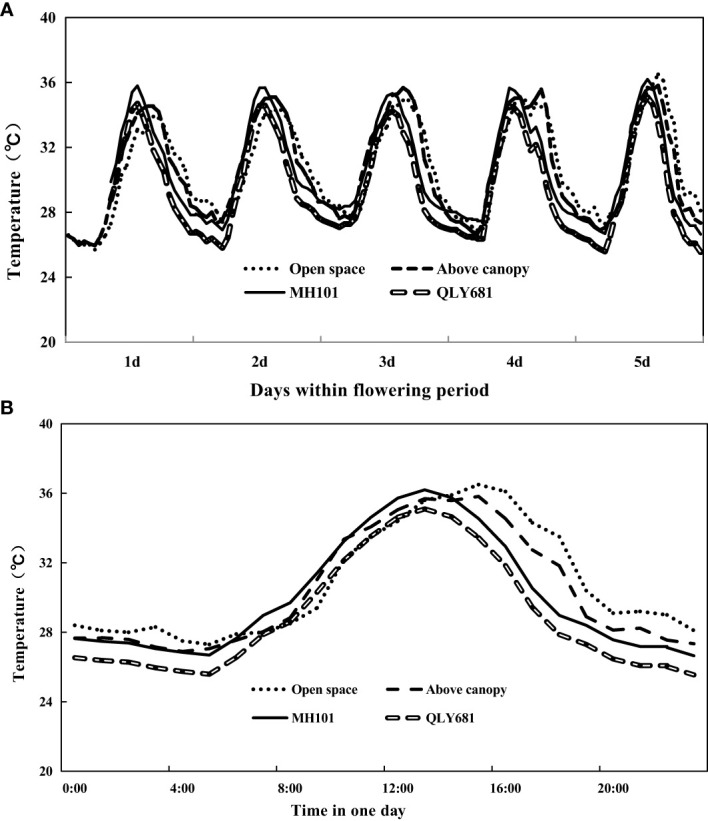
Monitoring crop canopy temperature (QLY681 and MH101), in comparison with the ambient temperature and the above canopy under the long-term hear scenario (LHS). Data was recorded from 5 August 2018 at 08 am to 9 August 2018 at 12 am. **(A)** continuous day-round temperature tendency in five days; **(B)** magnification of **(A)** during the day on 9 August.

The results of the canopy temperature of rice plants grown under LHS are shown in **Figure 3**. Our findings showed that at 10:00 (onset of daytime flowering) and at 12:00 (towards the end of daytime flowering) heat tolerant variety maintained lower canopy temperature compared to the above canopy temperature and open space temperature. Conversely, our findings showed that heat susceptible variety, had higher canopy temperature compared to above canopy temperature and open space temperature at 10:000 and 12:00. However, after 12:00 the canopy temperature of heat susceptible variety began to drop. We observed that at 20:00−when temperature had dropped to a relatively lower levels−both the heat tolerant and the heat susceptible variety maintained lower canopy temperature compared to open space and above canopy temperature. Our entire data showed that heat tolerant variety significantly maintained lower canopy temperature day and night throughout the entire flowering period, compared to heat susceptible variety. The differences observed in above canopy temperature and open space temperature during that period is also noteworthy. At 10:00 we observed higher above canopy temperature, compared to ambient temperature, but by 14:00−when daytime flowering had ended−ambient temperature began to increase and by 20:00 we observed higher ambient temperature compared to canopy temperature.

**Figure 4:**
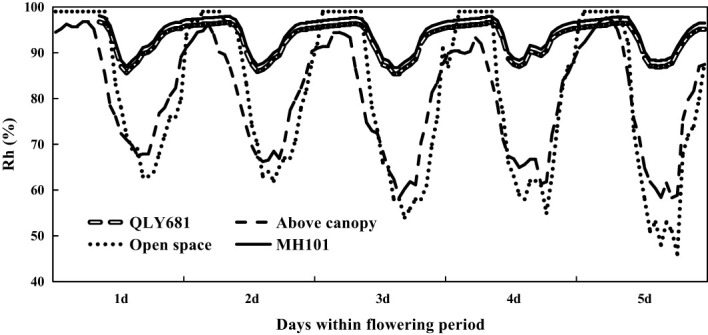
Monitoring relative humidity (Rh) within the rice canopy under a long-term heat scenario (LHS), in comparison with the ambient temperature and above the canopy.

In **Figure 4** we showed that heat tolerant variety had higher canopy relative humidity, compared to above canopy relative humidity and open space relative humidity. Similarly, we found that heat susceptible variety also had higher canopy relative humidity, compared to above canopy relative humidity and open space relative humidity. However, we observed that heat tolerant variety maintained a lower relative humidity, compared to heat susceptible variety throughout the entire period. Our findings also showed that above canopy relative humidity were mostly higher than open space relative humidity throughout the entire period.

**Figure 5:**
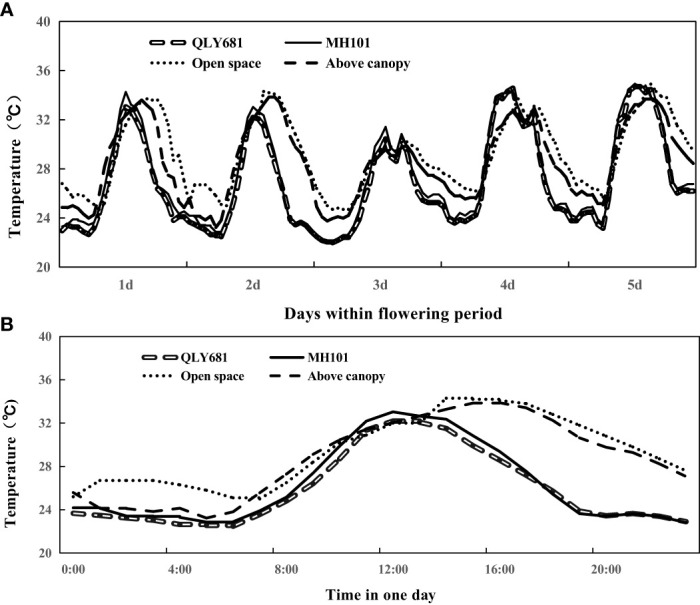
Monitoring crop canopy temperature (QLY681 and MH101) in comparison with the ambient and the above the canopy in a normal weather scenario. Data was recorded for five consecutive days during the flowering stage. **(A)** continuous day-round temperature tendency; **(B)** magnification of **(A)** on the day 26 August.

**Figure 6:**
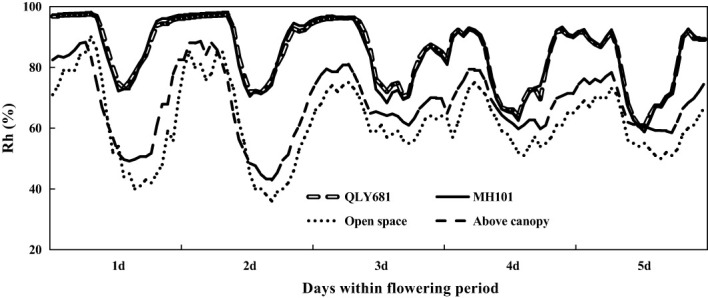
Monitoring relative humidity (Rh) within rice canopy under a normal weather scenario (NWS) in comparison with that in open space and above the canopy.

In **Figure 5** we revealed the results of the canopy temperature of rice grown under NWS. The results showed that the canopy temperature of the heat tolerant and heat susceptible rice varieties were similar throughout the entire period. Our results also showed that at 10:00 and 12:00, there were similarities in the canopy temperature of heat tolerant and heat susceptible rice varieties, compared to open space temperature and above canopy temperature. However, from 14:00 the canopy temperature of heat tolerant and heat susceptible varieties began to drop drastically and by 20:00 we observed significantly lower canopy temperature of heat tolerant and heat susceptible rice varieties, compared to open space temperature and above canopy temperature. We also observed differences in the open space and above canopy temperatures. At 20:00 we reported that open space temperature was higher than above canopy temperature, but at 10:00 and 14:00, they were similar. In **Figure 6**, we also observed that the canopy relative humidity of both the heat tolerant and heat susceptible varieties grown under NWS were similar throughout the entire period. However, we also observed that both the heat tolerant and heat susceptible rice varieties had higher canopy relative humidity compared to above canopy relative humidity and open canopy relative humidity. Our findings showed that above canopy relative humidity were mostly higher than open space relative humidity throughout the entire period.

### 3.5 Stomatal conductance under different heat regimes

Stomatal conductances were measured at 10:00 and 16:00 and our findings are shown in **Table 8**. Our results showed that heat tolerant varieties, SDWG005 and Quanliangyou 681, had higher stomatal conductance compared to heart susceptible varieties, Mianhui 101 and IR64.

**Table 8:** Stomatal conductance in four rice varieties under two weather scenarios during flowering under LHS and NWS.

Time	Variety	Stomatal conductance (mmol m^-2^ s^-1^)	
		LHS	NWS
10 am	Mianhui101	981.4 ± 71.6b**	563.8 ± 57.4b
	IR64	1003.8 ± 119.8b**	540.3 ± 48.0b
	Quanliangyou681	1137.5 ± 42.2a**	612.3 ± 31.5a
	SDWG005	1136.5 ± 101.7a**	599.3 ± 85.4a
4 pm	Mianhui101	335.3 ± 76.8c**	533.4 ± 20.7b
	IR64	400.8 ± 34.2b**	523.4 ± 54.4b
	Quanliangyou681	503.1 ± 75.1a*	571.1 ± 19.9a
	SDWG005	500.1 ± 86.5a*	574.6 ± 57.1a

** and * indicate significant differences at P<0.01 and P<0.05, respectively, between a variety in the long-term heat scenario (LHS) and the same variety under the normal weather scenario (NWS).

Different lowercase letters indicate significant differences between varieties under the same weather scenario at the P<0.05 level using Tukey’s test (n=15).

At 10:00 rice plants grown under LHS had the highest levels of stomatal conductance, while rice plant grown at NWS recorded the lowest levels in all four varieties. Conversely, at 16:00 we observed that rice plants grown in LHS had the lowest levels of stomatal conductance, while rice plants grown at NWS recorded the highest levels of stomatal conductance in all four varieties. Our results also showed that at 10:00 in all four varieties, there are significant difference−with Pvalue of <0.01−in stomatal conductance of NWS and LHS. However, at 16:00 we observed significant difference with Pvalue of <0.05 in stomatal conductance of NWS and LHS; except in Mianhui 101 and IR64 which had Pvalue of <0.01 between NWS and LHS.

## 4 Discussion

Advances in technology have improved the efficiency and reliability of micrometeorological monitoring in plants (Deery et al., 2016). In the 1990s, Chtioui et al. (1999) used micrometeorological data measured from 1993 to 1995 to estimate moisture occurrence at the flag leaf level of wheat and used generalised regression neural network to correctly predict 92.7% of the moisture duration periods critical to disease development. Liu and Xu (2018) noted that micometerologoical methods are the most commonly used to determine evapotranspiration in plants due to the high temporal resolution, large spatial observation scale, and relatively high accuracy. Yoshimoto et al. (2022); Cheabu et al. (2019) and Gong et al. (2023) have used micrometeorological monitoring to study the impact of heat stress on rice varieties.

Varietal heat tolerance screening with specific phenotypic traits and meteoro-physiological variables is paramount to heat tolerant breeding in rice. However, we do not know for sure which of the numerous phenotypic traits and meteoro-physiological variables could be reliable for screening of varietal heat tolerance in the field. In the present study, we used micrometeorological monitoring to determine varietal differences in rice grown under two different weather scenarios in heat –prone area; so as to form a reliable and effective method for varietal screening.

Michaletz et al. (2016) stated that canopy temperature of rice is controlled by changes in the environmental heat balance and heat conduction within the plant. Jiang et al. (2022) noted that leaves are the most sensitive organ for the short-term regulation of canopy temperature. The findings of this study showed that leaf temperature played some roles in regulating canopy temperature. Our results (**Tables 6**, **7**) reveal that from 10 am to 2 pm, heat susceptible varieties grown under LHS had higher leaf temperature, compared to heat tolerant varieties grown under the same scenario. However, within the same time frame, we observed no significant difference in the leaf temperature of heat susceptible and heat tolerant varieties grown under NWS. We also observed that both the heat tolerant and heat susceptible varieties had lower leaf temperature in NWS, compared to LHS. Our results showed that high leaf temperature correlated with high canopy temperature, while low leaf temperature resulted in low canopy temperature.

In *indica* rice varieties diurnal flower opening time, otherwise known as floret-opening time begin around 10 am (Wang et al., 2022; Wang et al., 2023). We reported that heat tolerant varieties grown under LHS maintained lower canopy temperature−not just during the crucial daytime flowering period of 10 am to 12 pm, but throughout the entire day and night−compared to heat susceptible varieties grown under the same scenario. Meanwhile, from 8 am to 2 pm−just before, during and just after flowering−we observed higher canopy temperature in heat susceptible varieties compared to ambient temperature, while in heat tolerant varieties we recorded lower canopy temperature compared to ambient temperature. After 2 pm we observed lower canopy temperature in both heat tolerant and heat susceptible varieties compared to the ambient temperature; however, heat tolerant varieties still maintained significantly lower temperature of 1 to 2 °C, compared to heat susceptible varieties throughout the day and night. Since rice leaves are the most sensitive organ responsible for short term regulation of canopy temperature, the results of our study suggest that the leaves of heat susceptible varieties are more sensitive to heat stress compared to the leaves of heat tolerant varieties. Therefore, the high sensitivity of the leaves of heat susceptible varieties to heat stress is likely the reason for their high canopy temperature. Our results showed that canopy temperature can be used to distinguish heat tolerant varieties from heat susceptible varieties in rice grown under heat stress. Furthermore, our findings also showed that canopy temperature can be used to screen for heat tolerance, not just during the flowering hours, but throughout the entire day and night.

In heat tolerant and heat susceptible varieties grown under NWS, we didn’t observe significant difference in their canopy temperature throughout the entire day, except between 8 am and 2 pm−just before, during and just after flowering−when heat tolerant variety had slightly higher temperature compared to heat susceptible varieties. These findings show that canopy temperature cannot be used as a distinguishing trait to screen for heat tolerance in rice plants grown under normal weather conditions.

Stomatal conductance is another trait associated with determining heat tolerance in rice. Stomatal conductace plays an important role in transpiration, which has been reported as an important process in canopy temperature regulation in rice (Jones, 1999; Yoshimoto et al., 2022). In **Table 8** we showed that at 10 am and 4 pm, heat tolerant varieties grown under LHS had higher stomatal conductance, compared to heat susceptible varieties grown under the same scenario. In other words, we observed higher stomatal conductance in heat tolerant varieties, compared to heat susceptible varieties throughout the entire day in rice plants grown under LHS. In heat tolerant varieties, increase stomatal conductance due to high temperature increased their transpiration rate. Higher transpiration can lead to increase in evaporative cooling, which ultimately transfers heat away from heat tolerant varieties. Consequently, the high stomatal conductance observed in heat tolerant varieties played significant roles in reducing their canopy temperature. Based on the results of this study, we can speculate that stomatal conductance can also be used to determine the level of heat tolerance in rice.

In this study we also monitored the micrometeorology of rice panicle. We reported that between 10 am and 2 pm, heat tolerant varieties grown under LHS had lower panicle temperature compared to heat susceptible varieties grown under the same scenario, but at 8 pm no significant difference was observed in both heat tolerant and heat susceptible varieties. However, when grown under NWS, we observed no significant difference throughout the entire day in the panicle temperature of both the heat tolerant and heat susceptible rice varieties. These findings suggest that panicle temperature can be used to screen for heat tolerance in rice grown under heat stress during the day. However, it’s not clear why there was no difference in their panicle temperature at night. We can speculate that the higher rate of evaporative cooling as a result of transpiration in the leaves during the day, played some roles in the significant difference in panicle temperature of heat tolerant and heat susceptible varieties observed between 10 am and 2 pm. Our findings also showed correlation between high canopy relative humidity and increase in panicle temperature. However, the correlations still didn’t explain why we observed no significant difference in panicle temperature at night. Therefore, there is need to carry out further studies to determine the reason for the similarities observed in the panicle temperature of both the heat tolerant and heat susceptible varieties at night.

Spikelet fertility can also be used to screen for heat tolerance in rice grown under heat stress, as it has been widely reported that high temperature had negative impact on them (Prasad et al., 2006; Tenorio et al., 2013; Huang et al., 2016; Prasanth et al., 2016; Cheabu et al., 2019). Spikelet fertility, otherwise known as seed-setting rate, is an important component of yield (Prasad et al., 2006). In this study, we also found that canopy temperature and panicle temperature affected spikelet fertility in rice. According to Zhang et al. (2016), heat stress causes more damage to spikelets than leaves of rice plants, due to the higher tissue temperatures in spikelets than in leaves. Yoshimoto et al. (2022) noted that evaluating heat induced spikelet sterility in rice requires researchers to account for micrometeorological differences between the ambient air above the rice canopy and conditions inside the plant canopy. The effect of canopy temperature and panicle temperature in mitigating heat induced spikelet sterility in rice plant is not yet well understood. We observed that from 10 am to 12 pm−crucial daytime flowering time window−heat tolerant varieties grown under LHS, had lower canopy temperatures and panicle temperature, compared to heat susceptible varieties grown under the same scenario. Consequently, we observed that between 10 am and 12 pm heat susceptible varieties grown under LHS, had significantly lower spikelet fertility rate, compared to heat tolerant varieties (**Table 4**). The results of this study showed that high canopy temperature and high panicle temperature decreased spikelet fertility in rice grown under heat stress. Similarly, we also found higher spikelet fertility in both the heat tolerant and the heat susceptible varieties grown under NWS, compared to those grown under LHS.

## 5 Conclusion

Varietal heat tolerance screening with specific phenotypic traits or meteoro-physiological variables is of crucial importance in rice breeding for heat tolerance. Micrometeorological monitoring is reliable way to ascertain the impact of heat stress in rice plants. The results presented in this study have shown that canopy temperature, panicle temperature and stomatal conductance can all be used to screen for heat tolerance in rice grown under heat stress; however, canopy temperature is the most reliable among them.

## Data availability statement

The original contributions presented in the study are included in the article/supplementary material. Further inquiries can be directed to the corresponding authors.

## Author contributions

WT: Data curation, Formal analysis, Investigation, Methodology, Software, Writing – original draft. QM: Data curation, Formal analysis, Investigation, Writing – original draft. YG: Data curation, Formal analysis, Investigation, Writing – original draft. YZ: Data curation, Formal analysis, Investigation, Writing – original draft. YW: Data curation, Formal analysis, Investigation, Writing – original draft. SD: Data curation, Formal analysis, Investigation, Writing – original draft. KA: Formal analysis, Software, Writing – review & editing. NO: Funding acquisition, Methodology, Supervision, Visualization, Writing – original draft, Writing – review & editing. XT: Conceptualization, Funding acquisition, Methodology, Supervision, Visualization, Writing – original draft, Writing – review & editing.

## References

[B1] Ayeneh A. Ginkel M. V. Reynolds M. P. Ammar K. (2002). Comparison of leaf, spike, peduncle and canopy temperature depression in wheat under heat stress. F. Crop Res. 79, 173–184. doi: 10.1016/S0378-4290(02)00138-7

[B2] Cheabu S. Panichawong N. Rattanametta P. Wasuri B. Kasemsap P. Arikit S.. (2019). Screening for spikelet fertility and validation of heat tolerance in a large rice mutant population. Rice Sci. 26, 229–238. doi: 10.1016/j.rsci.2018.08.008

[B3] Chtioui Y. Francl L. J. Panigrahi S. (1999). Moisture predictionfrom simple micrometeorological data. Phytopathology 89, 668–672. doi: 10.1094/PHYTO.1999.89.8.668 18944679

[B4] Conaty W. C. Mahan J. R. Neilsen J. E. Tan D. K. Y. Yeates S. J. Sutton B. G. (2015). The relationship between cotton canopy temperature and yield, fibre quality and water-use efficiency. F. Crop Res. 183, 329–341. doi: 10.1016/j.fcr.2015.08.010

[B5] Deery D. M. Rebetzke G. J. Berni J. A. J. James R. A. Condon A. G. Bovill W. D.. (2016). Methodology for high-throughput field phenotyping of canopy temperature using airborne thermography. Front. Plant Sci. 7, 1808. doi: 10.3389/fpls.2016.01808 27999580 PMC5138222

[B6] Dillahunty A. L. Siebenmorgen T. J. Mauromoustakos A. (2001). Effect of temperature, exposure duration, and moisture content on color and viscosity of rice. Cereal Chem. 78, 559–563. doi: 10.1094/CCHEM.2001.78.5.559

[B7] Dou Y. Huang R. Mansaray L. R. Huang J. (2019). Mapping high temperature damaged area of paddy rice along the Yangtze River using Moderate Resolution Imaging Spectroradiometer data. Int. J. Remote Sens. 41, 471–486.

[B8] Gong W. Proud C. Fukai S. Mitchell J. (2023). Low canopy temperature and high stomatal conductance contribute to high grain yield of contrasting japonica rice under aerobic conditions. Front. Plant Sci. 14, 1176156. doi: 10.3389/fpls.2023.1176156 37251759 PMC10214837

[B9] He L. Cleverly J. Wang B. Jin N. Mi C. (2018). Multi-model ensemble projections of future extreme heat stress on rice across southern China. Theor. Appl. Climatol. 133 (3–4), 1107–1118. doi: 10.1007/s00704-017-2240-4

[B10] Huang L. Sun Y. Peng S. Wang F. (2016). Genotypic Differences of Japonica Rice Responding to High Temperature in China. Agron. J. 108, 626–636.

[B11] Ishimaru T. Xaiyalath S. Nallathambi J. Sathishraj R. Jagadish K. S. V. (2015). Quantifying rice spikelet sterility in potential heat-vulnerable regions: field surveys in Laos and southern India. F. Crop Res. 190, 3–9. doi: 10.1016/j.fcr.2015.08.006

[B12] Jagadish S. V. K. Muthurajan R. Oane R. Wheeler T. R. Heuer S. Bennett J.. (2010). Physiological and proteomic approaches to address heat tolerance during anthesis in rice (*Oryza sativa L.*). J. @ Exp. Bot. 61, 143–156. doi: 10.1093/jxb/erp289 19858118 PMC2791117

[B13] Jiang M. Guo K. Wang J. Wu Y. Shen X. Huang L. (2022). Current status and prospects of rice canopy temperature research. Food Energy Secur. 12. doi: 10.1002/fes3.424

[B14] Jones H. G. (1999). Use of thermography for quantitative studies of spatial and temporal variation of stomatal conductance over leaf surfaces. Plant Cell Environ. 22, 1043–1055. doi: 10.1046/j.1365-3040.1999.00468.x

[B15] Li G. Zhang C. Zhang G. Fu W. Feng B. Chen T.. (2020). Abscisic acid negatively modulates heat tolerance in rolled leaf rice by increasing leaf temperature and regulating energy homeostasis. Rice Mar. 13, 13. doi: 10.1186/s12284-020-00379-3 PMC7070142 32170463

[B16] Liu S. Xu Z. (2018). “Micrometeorological methods to determine evapotranspiration,” in Observation and measurement. Ecohydrology. Eds. Li X. Vereecken H. (Springer, Berlin, Heidelberg).

[B17] Lopes M. S. Reynolds M. P. (2010). Partitioning of assimilates to deeper roots is associated with cooler canopies and increased yield under drought in wheat. Funct. Plant Bio. 37, 147–156. doi: 10.1071/FP09121

[B18] Lu G. Wu F. Wu W. Wang H. Zheng X. Zhang Y. (2014). Rice LTG1 is involved in adaptive growth and fitness under low ambient temperature. Plant J. 78, 468–480. doi: 10.1111/tpj.12487 24635058

[B19] Lu Z. Percy R. G. Qualset C. O. Zeiger E. (1998). Stomatal conductance predicts yields in irrigated Pima cotton and bread wheat grown at high temperatures. J. @ Exp. Bot. 49, 453–460. doi: 10.1093/jxb/49.Special_Issue.453

[B20] Lu Z. Radin J. W. Turcotte E. L. Percy R. Zeiger E. (1994). High yields in advanced lines of Pima cotton are associated with higher stomatal conductance, reduced leaf area and lower leaf temperature. Physiologia Pla. 92 (2), 266–272. doi: 10.1111/j.1399-3054.1994.tb05336.x

[B21] Maruyama A. Weerakoon W. M. W. Wakiyama Y. Ohba K. (2013). Effects of increasing temperatures on spikelet fertility in different rice cultivars based on temperature gradient chamber experiments. J. @ Agron. Crop Sci. 199 (6), 416–423. doi: 10.1111/jac.12028

[B22] Matsui T. Kagata H. (2003). Characteristics of floral organs related to reliable self-pollination in rice (Oryza sativaL.). Ann. Bot. 91 (4), 473–477. doi: 10.1093/aob/mcg045 12588727 PMC4241067

[B23] Matsui T. Kobayasi K. Yoshimoto M. Hasegawa T. (2007). Stability of rice pollination in the field under hot and dry conditions in the Riverina region of new south wales, Australia. Plant Prod. Sci. 10 (1), 57–63. doi: 10.1626/pps.10.57

[B24] Matsui T. Omasa K. (2002). Rice (*Oryza sativa L.*) varieties tolerant to high temperature at flowering: Anther characteristics. Ann. Bot. 89, 683–687. doi: 10.1093/aob/mcf112 12102523 PMC4233832

[B25] Matsui T. Omasa K. Horie T. (1997). High temperature-induced spikelet sterility of japonica rice at flowering in relation to air temperature, humidity and wind velocity conditions. Japanese J. Crop Sci. 66, 449–455. doi: 10.1626/jcs.66.449

[B26] Michaletz S. T. Weiser M. D. McDowell N. G. Zhou J. Kaspari M. Helliker B. R.. (2016). The energetic and carbon economic origins of leaf thermoregulation. Nat. Plants 2, 16129. doi: 10.1038/nplants.2016.129 27548589

[B27] Okpala N. E. Potcho M. P. An T. Ahator S. D. Duan L. Tang X. (2020). Low temperature increased the biosynthesis of 2-AP, cooked rice elongation percentage and amylose content percentage in rice. J. Cereal Sci. 93, 102980. doi: 10.1016/j.jcs.2020.102980

[B28] Okpala N. E. Potcho M. P. Imran M. An T. Bao G. He L.. (2021). Starch morphology and metabolomic analyses reveal that the effect of high temperature on cooked rice elongation and expansion varied in indica and japonica rice cultivars. Agronomy 11, 2416. doi: 10.3390/agronomy11122416

[B29] Perdomo J. A. Conesa M. A. Medrano H. Ribas-Carbó M. Galmés J. (2015). Effects of long-term individual and combined water and temperature stress on the growth of rice, wheat and maize: Relationship with morphological and physiological acclimation. Physiol. Plant 155, 149–165. doi: 10.1111/ppl.12303 25348109

[B30] Prasad P. V. V. Boote K. J. Allen L. H. Sheehy J. E. Thomas J. M. G. (2006). Species, ecotype and cultivar differences in spikelet fertility and harvest index of rice in response to high temperature stress. F. Crop Res. 95, 398–411. doi: 10.1016/j.fcr.2005.04.008

[B31] Prasanth V. V. Basava K. R. Babu M. S. Venkata Tripura V. G. N. Devi S. J. S. R. Mangrauthia S. K.. (2016). Field level evaluation of rice introgression lines for heat tolerance and validation of markers linked to spikelet fertility. Physiol. Mol. Bio. Plants 22, 179–192. 27436910 10.1007/s12298-016-0350-6 PMC4938818

[B32] Satake T. Yoshida S. (1978). High temperature-induced sterility in indica rices at flowering. Japanese J. Crop Sci. 47 (1), 6–17. doi: 10.1626/jcs.47.6

[B33] Sathishraj R. Bheemanahalli R. Ramachandran M. Dingkuhn M. Muthurajan R. Krishna J. S. V. (2016). Capturing heat stress induced variability in spikelet sterility using panicle, leaf and air temperature under field conditions. F. Crop Res. 190, 10–17. doi: 10.1016/j.fcr.2015.10.012

[B34] Shi W. Ishimaru T. Gannaban R. B. Oane W. Jagadish S. V. K. (2015). Popular rice (L.) cultivars show contrasting responses to heat stress at gametogenesis and anthesis. Crop Sci. 55 (2), 589. doi: 10.2135/cropsci2014.01.0054

[B35] Shi W. Yin X. Struik P. C. Solis C. Xie F. Schmidt R. C.. (2017). High day- and night-time temperatures affect grain growth dynamics in contrasting rice genotypes. J. Exp. Bot. 68, 5233–5245. doi: 10.1093/jxb/erx344 29106621 PMC5853565

[B36] Siebert S. Ewert F. Eyshi Rezaei E. Kage H. Graß R. (2014). Impact of heat stress on crop yield—on the importance of considering canopy temperature. Environ. Res. Let. 9 (4), 044012. doi: 10.1088/1748-9326/9/4/044012

[B37] Sun Q. Zhao. Y. Zhang Y. Chen S. Ying Q. Lv Z.. (2022). Heat stress may cause a significant reduction of rice yield in China under future climate scenarios. Sci. Total Environ. 818, 151746. doi: 10.1016/j.scitotenv.2021.151746 34801492

[B38] Takai T. Yano M. Yamamoto T. (2010). Canopy temperature on clear and cloudy days can be used to estimate varietal differences in stomatal conductance in rice. F. Crop Res. 115 (2), 165–170. doi: 10.1016/j.fcr.2009.10.019

[B39] Teixeira E. I. Fischer G. van Velthuizen H. Walter C. Ewert F. (2013). Global hot-spots of heat stress on agricultural crops due to climate change. Agric. For Met. 170, 206–215. doi: 10.1016/j.agrformet.2011.09.002

[B40] Tenorio F. A. Ye C. Redoñ E. Sierra S. Laza M. Argayoso M. A. (2013). Screening rice genetic resources for heat tolerance. SABRAO J. Breeding Genet. 45 (3), 371–381.

[B41] Tian X. Luo H. Zhou H. (2009). Research on heat stress of rice in China: Progress and prospect. Chin. Agric. Sci. Bull. (in Chinese). 25 (22), 166–168.

[B42] Tian X. Matsui T. Li S. Yoshimoto M. Kobayasi K. Hasegawa T. (2010). Heat-induced floret sterility of hybrid rice (*Oryza sativa L.*) cultivars under humid and low wind conditionsin the field of Jianghan Basin, China. Plant Prod. Sci. 13 (3), 243–251. doi: 10.1626/pps.13.243

[B43] Wang M. Chen M. Huang Z. Zhou H. Liu Z. (2023). Advances on the study of diurnal flower-opening times of rice. Int. J. Mol. Sci. 224, 10654. doi: 10.3390/ijms241310654 PMC10341841 37445832

[B44] Wang M. Zhu X. Peng G. Liu M. Zhang S. Chen M.. (2022). Methylesterification of cell-wall pectin controls the diurnal flower-opening times in rice. Mol. Plant 15, 956–972. doi: 10.1016/j.molp.2022.04.004 35418344

[B45] Wassman R. Jagadish S. V. K. Sumfleth K. Pathak H. Howell G. Ismail A.. (2009). Chapter 3. Regional vulnerability of climate change impacts on Asian rice production and scope for adaptation. Adv. Agron. 102, 91–133. doi: 10.1016/S0065-2113(09)01003-7

[B46] Yamori W. Sakata N. Suzuki Y. Shikanai T. Makino A. (2011). Cyclic electron flow around photosystem I via chloroplast NAD(P)H dehydrogenase (NDH) complex performs a significant physiological role during photosynthesis and plant growth at low temperature in rice. Plant J. 68, 966–997. doi: 10.1111/j.1365-313X.2011.04747.x 21848656

[B47] Yan H. Zhang B. Zhang Y. Chen X. Xiong H. Matsui T and Tian X. (2017). High temperature induced glume closure resulted in lower fertility in hybrid rice seed production. Front. Plant Sci. 7, 1960. doi: 10.3389/fpls.2016.01960 28105031 PMC5214948

[B48] Yoshimoto M. Fukuoka M. Hasegaw T. Utsumi M. Ishigooka Y. Kuwagata T. (2011). Integrated micrometeorology model for panicle and canopy temperature (IM2PACT) for rice heat stress studies under climate change. J. @ Agric. Met. 67 (4), 233–247. doi: 10.2480/agrmet.67.4.8

[B49] Yoshimoto M. Fukuoka M. Tsujimoto Y. Matsui T. Kobayasi K. Saito K.. (2022). Monitoring canopy micrometeorology in diverse climates to improve the prediction of heat-induced spikelet sterility in rice under climate change. Agric. For. Meteorol. 316, 108860. doi: 10.1016/j.agrformet.2022.108860

[B50] Zhang C. X. Feng B. H. Chen T. T. Fu W. M. Li H. B. Li G. Y.. (2018). Heat stress-reduced kernel weight in rice at anthesis is associated with impaired source-sink relationship and sugars allocation. Environ. Exp. Bot. 155, 718–733. doi: 10.1016/j.envexpbot.2018.08.021

[B51] Zhang C. X. Fu G. F. Yang X. Q. Yang Y. J. Zhao X. Chen T. T.. (2016). Heat stress effects are stronger on spikelets than on flag leaves in rice due to differences in dissipation capacity. J. @ Agron. Crop Sci. 202 (5), 394–408. doi: 10.1111/jac.12138

